# Online real-time mass spectrometry-driven dynamic monitoring and optimal endpoint prediction schema development for herbal medicine decoction process

**DOI:** 10.1186/s13020-026-01377-1

**Published:** 2026-03-16

**Authors:** Junxian Wu, Liping Kang, Yutong Hua, Xiang Li, Zidong Qiu, Luqi Huang

**Affiliations:** 1 School of Pharmacy, Chengdu University of Traditional Chinese Medicine, Chengdu, 611137 China; 2 State Key Laboratory for Quality Ensurance and Sustainable Use of Dao-Di Herbs, National Resource Center for Chinese Materia Medica, China Academy of Chinese Medical Sciences, Beijing, 100700 China

**Keywords:** Online real-time mass spectrometry, Dynamic monitoring, Decoction endpoint, Zhenwu Decoction, Toxicity attenuation and efficacy preservation

## Abstract

**Background:**

The decoction process of compound formulae is a critical procedure in traditional herbal medicine for reducing toxicity and preserving efficacy. However, due to the high complexity of herb ingredients and chemical constituents in the formulae, precise real-time monitoring of the dynamic changes in the decoction system and accurately predicting its optimal endpoint remain significant challenges that current technologies struggle to address.

**Purpose:**

To develop an online real-time mass spectrometry (ORT-MS)-driven strategy for real-time monitoring complex components during the decoction of compound formulae, and to establish a validated schema for predicting the optimal decoction endpoint, using Zhenwu Decoction as a case study.

**Results:**

A novel ORT-MS monitoring device was established successfully, enabling continuous long-term (> 240 min) MS monitoring of various compounds in Zhenwu Decoction. By quantitatively tracking the decoction profiles of key toxic (e.g., hypaconitine) and bioactive (e.g., benzoylhypaconitine) compounds, a method for evaluating overall toxicity/bioactivity of the decoction was built. Furthermore, a comprehensive schema for determining the optimal decoction endpoint was proposed, integrating five-dimension: safety, bioactivity, time, energy consumption, and financial cost (SBTEF). The results demonstrated, under the experimental conditions and dosage adopted in this study, that the optimal decoction endpoint for Zhenwu Decoction is about 150 min when all factors are considered.

**Conclusion:**

This study proposes a universal MS-driven real-time analysis strategy and endpoint prediction schema for herbal medicine decoction process, providing a novel scientific tool for decoction quality evaluation (especially toxicity attenuation/efficacy preservation) to promote herbal medicine quality and rational clinical use.

**Supplementary Information:**

The online version contains supplementary material available at 10.1186/s13020-026-01377-1.

## Introduction

The co-decoction of multiple herbal medicine after compound compatibility is the main form of clinical medication in traditional medicine [1]. The decoction process involves complex physical and chemical changes, such as the dynamic dissolution and mutual transformation of multiple components. These dynamic changes constitute the material basis for the efficacy and toxicity of the compound, and determine the exertion of efficacy and medication safety. Specifically manifested as: (1) The dissolution and synergistic transformation of active constituents. For instance, after 2–4 h of decoction of Asian ginseng and American ginseng, the content of rare ginsenosides, which are more beneficial to human health, will increase significantly [2]; (2) Reduction and transformation of toxic components: A typical example is the highly toxic diester-type alkaloids (e.g., aconitine) present in Aconitum. These alkaloids can undergo hydrolysis to reduce toxicity when subjected to thorough decoction, converting into less toxic monoester-type alkaloids and non-toxic aminoalcohol-type alkaloids [3]. Therefore, analyzing the changes in chemical components during the decoction process of compound herbal medicine not only helps explore the rules of compatibility of herbal medicine and elucidate their compatibility mechanism, but also provides a material basis for the scientific judgment of the decoction endpoint of herbal medicine, thereby ensuring medication safety and stable efficacy from the source.

In recent years, a variety of analytical techniques have been applied to the study of components during the decoction process of Chinese medicine compounds [4, 5], facilitating the shift of herbal medicine decoction from traditional experience-based guidance to theoretical research. However, the decoction system of herbal medicine compounds is extremely complex, and traditional analytical methods have limitations in investigating the decoction process, especially in predicting the decoction endpoint. Spectroscopic techniques (e.g., UV–visible spectroscopy) [6], Fourier transform infrared spectroscopy [7] and nuclear magnetic resonance spectroscopy [8] exhibit notable limitations when analyzing complex systems like herbal medicine compounds. Relying on characteristic functional group absorption signals, they suffer from signal overlap (due to hundreds of components) and strong background interference from matrix components (carbohydrates, amino acids, inorganic salts), hindering accurate target component identification. Commonly used mass spectrometry techniques, such as Ultra-performance liquid chromatography-tandem quadrupole time-of-flight mass spectrometry (UPLC-Q-TOF–MS/MS) [9] or electrospray mass spectrometry (ESI–MS) [10], are superior to spectroscopic techniques in terms of component identification accuracy. They can perform component identification and content determination on samples, and provide information on the overall changes of chemical components. However, critical limitations remain. On the one hand, most of these methods rely on a static comparative research schema of “pre-decoction vs. post-decoction”, focusing on the component analysis of end products. They lack dynamic analysis from a temporal dimension, making it impossible to comprehensively evaluate and predict the optimal decoction endpoint. On the other hand, these methods require complex pretreatment of samples (such as extraction and filtration), which easily leads to the loss of target components or changes in their chemical properties. Additionally, the offline processing and detection modes make it difficult to capture the dynamic changes of components in real time and reveal the rules governing the formation and consumption of reaction intermediates, which limits the in-depth understanding of the reaction mechanism of compound decoction. The optimal decoction endpoint is a core key to achieving “toxicity attenuation and efficacy preservation” for herbal medicine compounds, as it directly determines the optimal efficacy and potential toxicity of the decoction. The limitations of traditional analytical methods in dynamic tracking, endpoint prediction, and elimination of complex matrix interference make herbal medicine decoction difficult to break away from reliance on experience, and it is impossible to accurately balance efficacy and safety. Therefore, there is an urgent need to develop analytical strategies that can overcome the above limitations. Real-time mass spectrometry technology, with its advantages of high-specificity recognition, high-sensitivity detection, no need for complex pretreatment, and real-time dynamic tracking, can not only reveal the laws of component changes but also provide core technical support for the prediction of decoction endpoints.

Zhenwu Decoction (ZWD) is a classic prescription recorded in Treatise on Febrile Diseases (Shanghan Lun, an ancient and authoritative TCM classic), composed of Aconiti lateralis Radix praeparata (Heishunpian), Paeoniae Radix Alba (Baishao), Poria (Fuling), Zingiberis Rhizoma Recens (Shengjiang), and Atractylodis Macrocephalae Rhizoma (Baizhu). It is mainly indicated for heart failure caused by heart-kidney yang deficiency and internal retention of water-dampness [11, 12]. As the monarch drug, Fuzi not only contains toxic components such as aconitine (a diester-type alkaloid) that require close attention [13], but also contains active monoester-type and amino alcohol-type components (e.g., benzoylaconine and aconine) produced by hydrolysis, which exert cardiotonic and anti-inflammatory effects. In addition, glycosides such as paeoniflorin (from Baishao), triterpenoids (from Fuling), and gingerols (from Shengjiang) in prescription are also important active substances of ZWD, which synergistically exert the effects of warming yang and the promoting diuresis (a core herbal medicine therapeutic effect). The dynamic increase and decrease of toxic components and active ingredients in ZWD exactly serve as an ideal method for investigating “toxicity attenuation and efficacy preservation” during the decoction process and the scientific determination of the decoction endpoint. In view of this, this study takes ZWD as the research object, utilizes a self-constructed real-time analysis device for the herbal medicine decoction system, and establishes a real-time mass spectrometry-driven research strategy for the decoction process of herbal medicine compounds. Meanwhile, it develops an application demonstration using the mechanism of “toxicity attenuation and efficacy preservation” during the decoction of ZWD as a template. By capturing the characteristics of component changes at different decoction stages and quantitatively tracking the decoction profiles of key toxic (e.g., hypaconitine) and bioactive (e.g., benzoylhypaconitine) compounds, this study aims to provide a new perspective for scientifically explaining herbal medicine decoction theory, optimizing decoction processes, scientifically predicting herbal medicine decoction endpoints, and analyzing the complex mechanism of compound decoction.

## Materials and methods

### Reagents, chemicals and materials

Fuzi, Baizhu, Baishao, and Fuling were all purchased from Anhui Haosen Pharmaceutical Co., Ltd. These herbs were crushed and sieved through a 0.15 mm (100-mesh) sieve to obtain uniform sample powders, which were used for subsequent experiments. Fresh Shengjiang was purchased from a local supermarket (Beijing, China), and cut into uniform thin slices (2.00 ~ 3.00 mm). All specimens were identified by Prof. Liping Kang, stored at the National Resource Center for Chinese Materia Medica, Chinese Academy of Chinese Medical Sciences (specimen number: ZZFZ2025001, ZZFL2025001, ZZSJ2025001, ZZBZ2025001, ZZBS2025001). The reference standards (paeoniflorin and benzoylmesaconitine) were provided by Beijing Beite Renkang Biomedical Technology Co., Ltd. (purity > 98%). Both standards were dissolved in water to prepare standard stock solutions with a concentration of 1.00 mg/mL. The water used in the experiment was Watsons purified water (Guangzhou, China).

### Establishment of a real-time mass spectrometry monitoring platform for herbal medicine compound decoction

A monitoring platform named online real-time mass spectrometry (ORT-MS) was independently established for herbal medicine compound decoction. The platform is mainly divided into three functional units: (1) Compound decoction unit: a 500 mL three-necked flask, equipped with a digital temperature-controlled magnetic heating mantle (TAISITE, 98-Ⅱ-CN) and a reflux condensing tube; (2) Sample transmission unit: including inert silicone tubing (inner diameter: 2 mm), a multi-channel peristaltic pump (LongerPump, BT100-3J), and a 0.45 µm polyethersulfone filter (Jinteng, Tianjin, China); (3) Real-time mass spectrometry detection unit: a self-made spray nozzle coupled with a linear ion trap mass spectrometer (Thermo Fisher Scientific, Massachusetts, USA).

### Optimization of key parameters

Prior to the analysis of actual samples, the key parameters of ORT-MS need to be systematically optimized. Using the ion peak intensities of the main components (paeoniflorin in negative ion mode and benzoylmesaconitine in positive ion mode) as indicators, a combination of automatic and manual adjustment was adopted: firstly, the capillary voltage and tube lens parameters were automatically optimized by the instrument; subsequently, the spray voltage, spray gas pressure, and capillary temperature were manually optimized. The optimization process employed a single-factor optimization strategy: the capillary temperature was systematically adjusted within the range of 100–450 ℃ to determine the temperature with the optimal signal response; then, the optimal capillary voltage was determined through tests within the range of 3.0–6.0 kV; finally, the spray gas pressure was optimized within the range of 0.2–0.5 MPa to confirm the optimal pressure parameters.

### Real-time monitoring of the decoction process for ZWD

Each herb was accurately weighed: 0.414 g of Fuzi, 0.276 g of Baizhu, 0.414 g Baishao, 0.414 g of Fuling, and 0.414 g of Shengjiang. The herbs were placed in a 500 mL three-necked flask, followed by the addition of 400 mL ultrapure water to soak for 30 min. The decoction sample was prepared with each herb at a solid‑to‑liquid ratio of about 1:1000, an extremely low concentration that minimized matrix effects and mutual interference among different components. The decoction process was divided into two stages: in the initial stage, the mixture was heated over high heat until boiling, then switched to low heat to maintain a gentle boiling state, with the experiment continuously recorded for 240 min. To ensure the reliability of the results, three independent replicate experiments were conducted in positive ion mode and negative ion mode, respectively. To evaluate the interactions between herbs, each single herb was decocted and subjected to subsequent analysis operations strictly according to its corresponding concentration in the compound.

### ORT-MS data processing

The online mass spectrometry system acquires full-scan data (m/z 100–1500), and can obtain approximately 72,000 signal intensity values for each chemical component within 240 min. After the dynamic signals are denoised using the boxcar smoothing algorithm (points = 3), the extracted ion chromatograms (EICs) of the target compounds are extracted. MATLAB R2016a software with its built-in curve fitting tool is used to fit the data based on the Gaussian model and Fourier model, thereby achieving the visual analysis of the dynamic changes in chemical components during the decoction process.

### Construction of the five-dimensional SBTEF decoction endpoint prediction schema

To extend the research conclusions of ZWD to more herbal medicine and establish universal criteria for determining the decoction endpoint, this study propose a comprehensive five-dimensional predictive schema. The schema is constructed based on five core dimensions: safety, bioactivity, time, energy consumption, and financial cost (SBTEF). By clarifying the weight assignment of each dimension, objective and standardized determination of the decoction endpoint is achieved. For the safety dimension, the primary criterion is that the content of toxic components must be maintained within the safety threshold; in terms of the bioactivity dimension, the key indicator requires that active components be as abundant as possible on the premise of safety and be stably maintained in the high-efficacy range; regarding the time dimension, the core requirement is to shorten the decoction duration and simplify the sample pretreatment process; as for the energy consumption, the key measurement standard is to reduce the use of energy, mitigate the potential environmental hazards, and thus achieve the goal of greening the research process; for the cost-effectiveness dimension, the overall research cost is controlled by calculating reagent consumption and labor input.

## Results and discussion

### Development and parameter optimization of ORT-MS

**Fig. 1:**
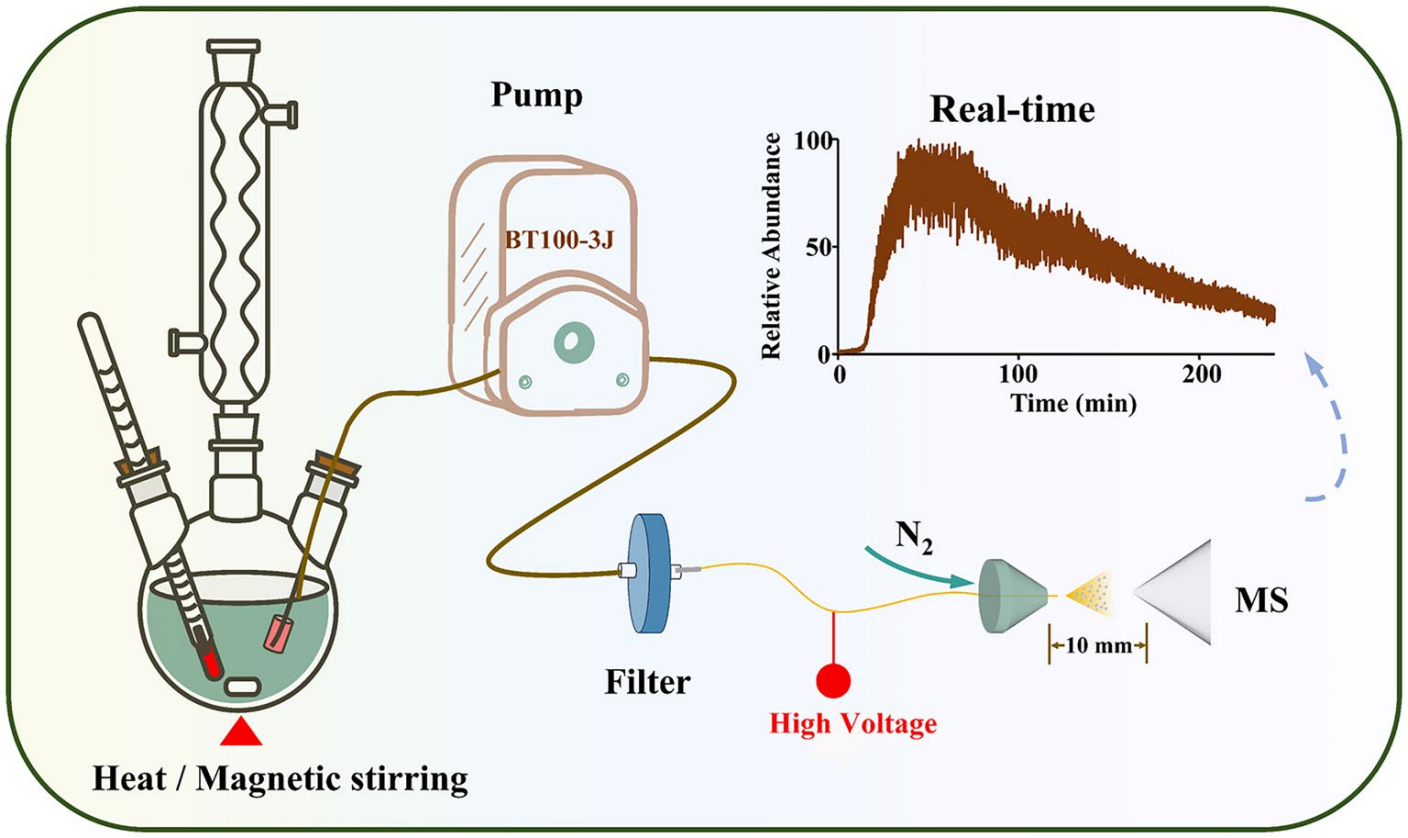
Schematic illustration of ORT-MS device

Firstly, a real-time mass spectrometry-based monitoring platform for herbal compound decoctions was developed and named ORT-MS, featuring a simple structure, easy operability, high versatility, and excellent stability. As shown in Fig. 1, this platform was constructed by coupling an LTQ XL linear ion trap mass spectrometer with a self-made ESI ion source. On the left is the reaction unit, consisting of a three-necked flask, a temperature-controlled magnetic heating mantle, and a condenser tube: the three-necked flask is used to hold herbs and solvents; the temperature-controlled magnetic heating mantle realizes heating and magnetic stirring; the condenser tube prevents fluctuations in compound concentration caused by solvent volatilization. The sample transmission channel is an inert silicone tube (inner diameter: 2.00 mm), with its inlet wrapped in non-woven fabric to avoid pipeline blockage. A precision peristaltic pump provides a constant flow rate, ensuring the stability and repeatability of detection signals. Additionally, the 0.45 μm microporous membrane achieves online impurity removal, and when combined with the high ionization efficiency of the self-made ESI ion source, it enables direct injection analysis of samples with complex matrices without any offline pretreatment, significantly shortening the detection cycle. This integrated design thereby realizes the real-time and accurate detection of chemical components during the decoction of herbal compound decoctions. Compared with existing mass spectrometry monitoring systems such as ND-APCI-MS [14] and PSI-mini-MS [15], ORT-MS enables continuous, uninterrupted sampling and real-time ionization analysis of complex decoction solutions from traditional Chinese medicine (TCM). It accomplished 72,000 mass spectrometry signal acquisitions within 240 min, demonstrating an irreplaceable capability for the real-time multi-component monitoring of complex TCM compound decoction systems.

**Fig. 2:**
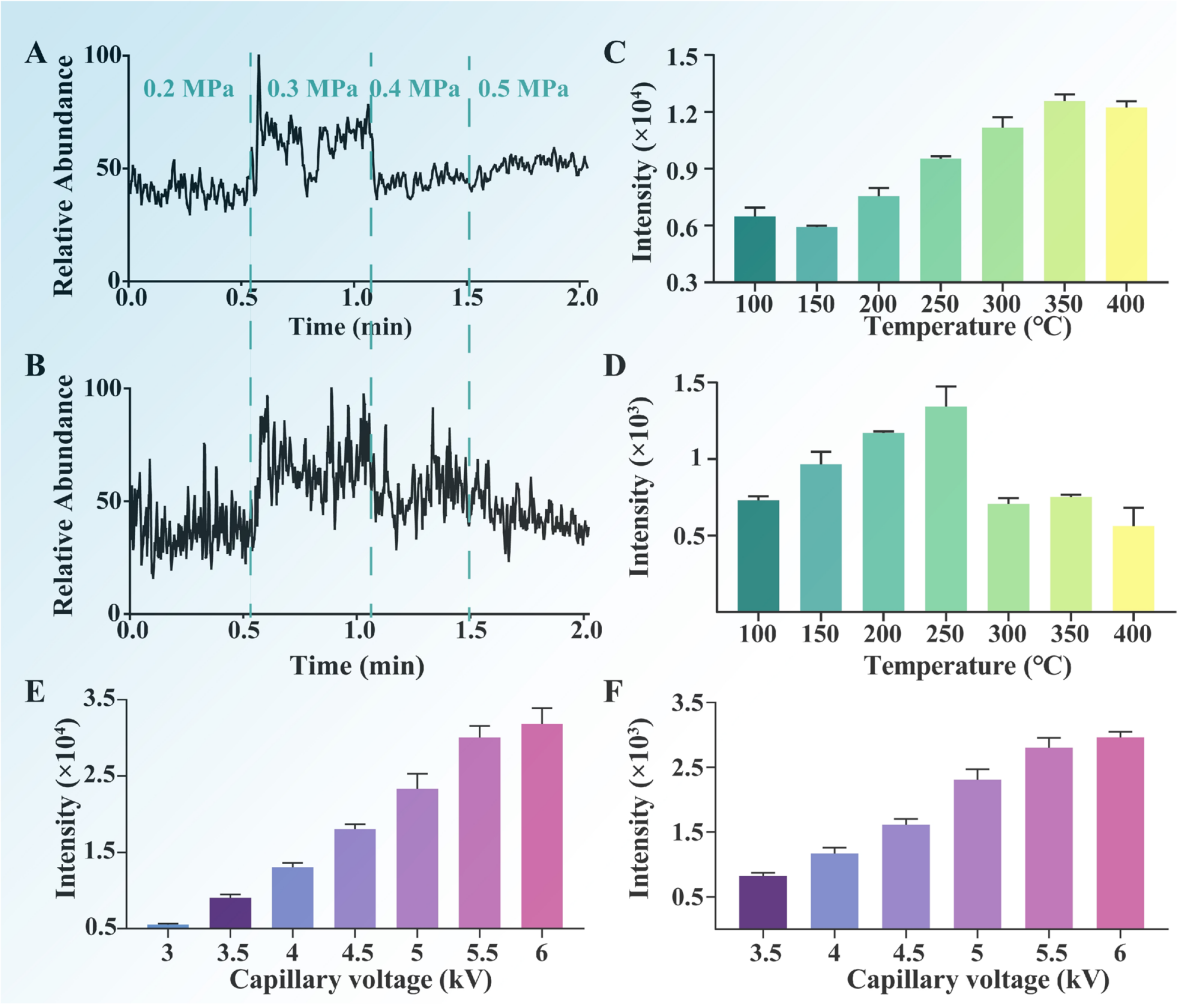
**A**, **B** TIC spectra for optimizing ionization spray gas pressure in positive and negative ion modes, respectively. **C**, **D** Signal intensities of the target analyte obtained during optimization of capillary temperature in positive and negative ion modes, respectively. **E**, **F** Signal intensities of the target analyte obtained during optimization of capillary voltage in positive and negative ion modes, respectively

To achieve more efficient ionization and detection, the key parameters of ORT-MS (spray voltage, spray gas pressure, and capillary temperature) were systematically optimized based on the signal intensities of the key components (paeoniflorin and benzoylmesaconitine) (Fig. 2). As shown in Fig. 2A, B, when high-purity nitrogen was used as the spray gas, the total ion current (TIC) signal intensity reached the maximum at a spray gas pressure of 0.3 MPa in both positive and negative ion modes; thus, the spray gas pressure was set to 0.3 MPa for both modes. In positive ion mode (Fig. 2C), the signal response of benzoylmesaconitine increased with the rise in capillary temperature within the range of 100–350 ℃, but slightly decreased when the temperature was between 350 and 400 ℃. Therefore, the capillary temperature for positive ion mode was determined to be 350 ℃. In negative ion mode (Fig. 2D), paeoniflorin exhibited the highest signal response at 250 ℃, so 250 ℃ was selected as the capillary temperature for negative ion mode. Further optimization of the spray voltage revealed that the TIC signal intensity gradually increased with the increase in voltage. Within the range of 5.5–6.0 kV, the difference in compound signal responses was minimal; however, excessively high voltage was prone to causing discharge phenomena. Based on this, the spray voltage for positive ion mode was set to 5.5 kV (Fig. 2E), and the corresponding spray voltage for negative ion mode was set to − 5.5 kV (Fig. 2F). These optimized parameters collectively ensured efficient ionization and stable detection of key components in herbal compound decoctions, laying a reliable foundation for the real-time monitoring performance of the ORT-MS platform.

### Analytical performance of ORT-MS for key components in ZWD

**Fig. 3:**
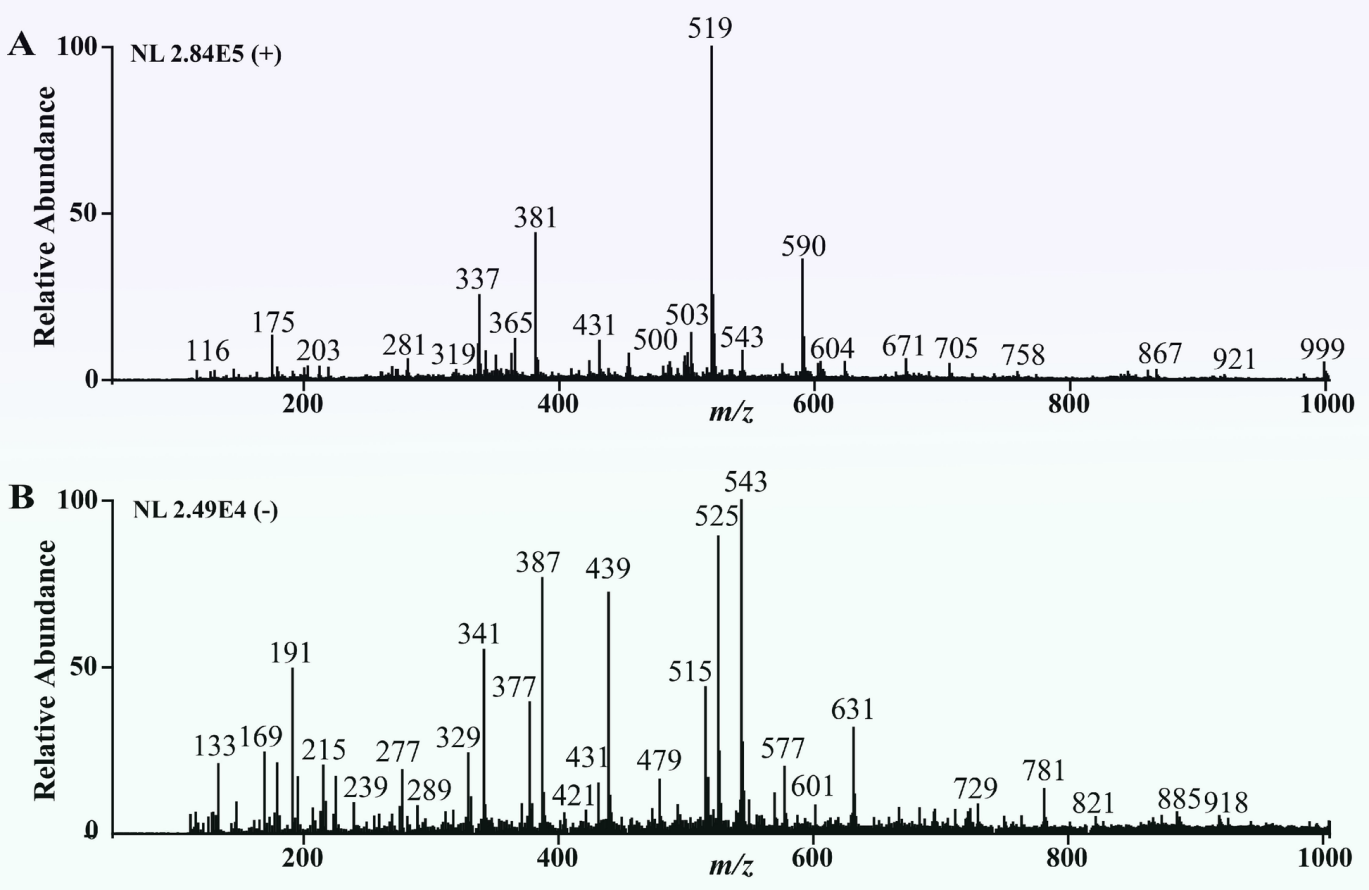
The ORT-MS fingerprinting of ZWD in positive ion mode (**A**) and negative ion mode (**B**)

After condition optimization, the real-time mass spectrometry monitoring platform for herbal medicine compound decoction established in this study exhibits the capability to detect various target components. This platform enables the continuous, sensitive collection of MS data from herbal medicine compound decoction samples; for target components of different structural types, it can acquire clear, accurate mass spectra and facilitate their precise structural identification using tandem MS data. The results showed that alkaloid components from Fuzi (e.g., benzoylmesaconitine, m/z 590; benzoylaconitine, m/z 604) exhibited significant responses in positive ion mode (Fig. 3A) with high ionization efficiency. In contrast, characteristic monoterpene glycoside components from Baishao (e.g., paeoniflorin, m/z 525; paeoniflorin sulfite, m/z 543) displayed stronger responses in negative ion mode (Fig. 3B). Based on this, a dual ion scanning mode was adopted in this study to conduct comprehensive analysis of ZWD decoction samples under positive and negative ion conditions, respectively, effectively covering multiple classes of chemical components in the compound decoction. By comprehensively comparing standard reference data, MS/MS fragmentation patterns reported in the literature, and systematically searching professional databases including ChemBank (http://chembank.med.harvard.edu/) and MassBank (http://www.massbank.jp/), a total of 51 target components were identified. These components cover key compound categories such as alkaloids and monoterpene glycosides, including 24 alkaloids derived from Fuzi. Key parameters of all identified compounds, such as their names, molecular formulas, molecular weights, and characteristic fragment ions, are listed in Table S1.

### Mechanism elucidation of herbal compatibility for attenuating toxicity and preserving efficacy during ZWD decoction based on ORT-MS

#### Dynamic changes of the main components in Fuzi during the decoction process

Fuzi is the lateral root of *Aconitum carmichaelii* Debx., possessing a variety of pharmacological activities, including analgesic, anti-inflammatory, immunomodulatory, antidepressant, and antitumor effects [16, 17], as well as a notable cardiotonic effect. However, the alkaloids in Fuzi serve not only as the primary material basis for its pharmacological activities but also as an important source of its toxicity [18]. Research has confirmed that diester-type alkaloids, which contain two ester bonds (an acetyl group at the C-8 position and a benzoyl group at the C-14 position) in their structure, exhibit strong cardiotoxicity and neurotoxicity, and are the primary substances responsible for clinical toxic reactions induced by Fuzi [19, 20]. Under heating conditions, the acetyl group at the C-8 position of these alkaloids is prone to hydrolysis and further degrading into monoester-type aconitines [15]. As key active components in Fuzi, monoester-type aconitines (e.g., benzoylhypacoitine, benzoylmesaconine, benzoylaconitine) have reduced toxicity (only 1/200–1/500 that of diester-type aconitines) due to the decreased number of ester bonds [21], Moreover, they have clear pharmacological effects, such as significantly enhancing myocardial contractility and improving cardiac function, and serve as the core material basis for Fuzi to exert its traditional efficacy of “Restoring Yang to Rescue from Collapse”. If heating conditions persist, the acyl group at the C-14 position of monoester-type alkaloids undergoes further hydrolysis to form aminoalcohol-type alkaloids. This hydrolysis process results in a further decrease in toxicity (only 1/2,000–1/4,000 that of aconitine), but their pharmacological activities correspondingly diminish [22]. Thus, during the transformation of Fuzi alkaloids, a higher content of monoester-type alkaloids results in stronger efficacy; if the transition stage of transformation is characterized by low levels of monoester-type alkaloids and high levels of aminoalcohol-type alkaloids, it will lead to a decrease in the overall efficacy of the system. Therefore, appropriate time control and herbal medicine compound compatibility are crucial for promoting the production of more monoester-type alkaloids. This rule not only clarifies the core correlation between Fuzi’s efficacy and alkaloid types but also provides a key basis for the process optimization of “toxicity attenuation and efficacy preservation” in the clinical application of Fuzi.

**Fig. 4:**
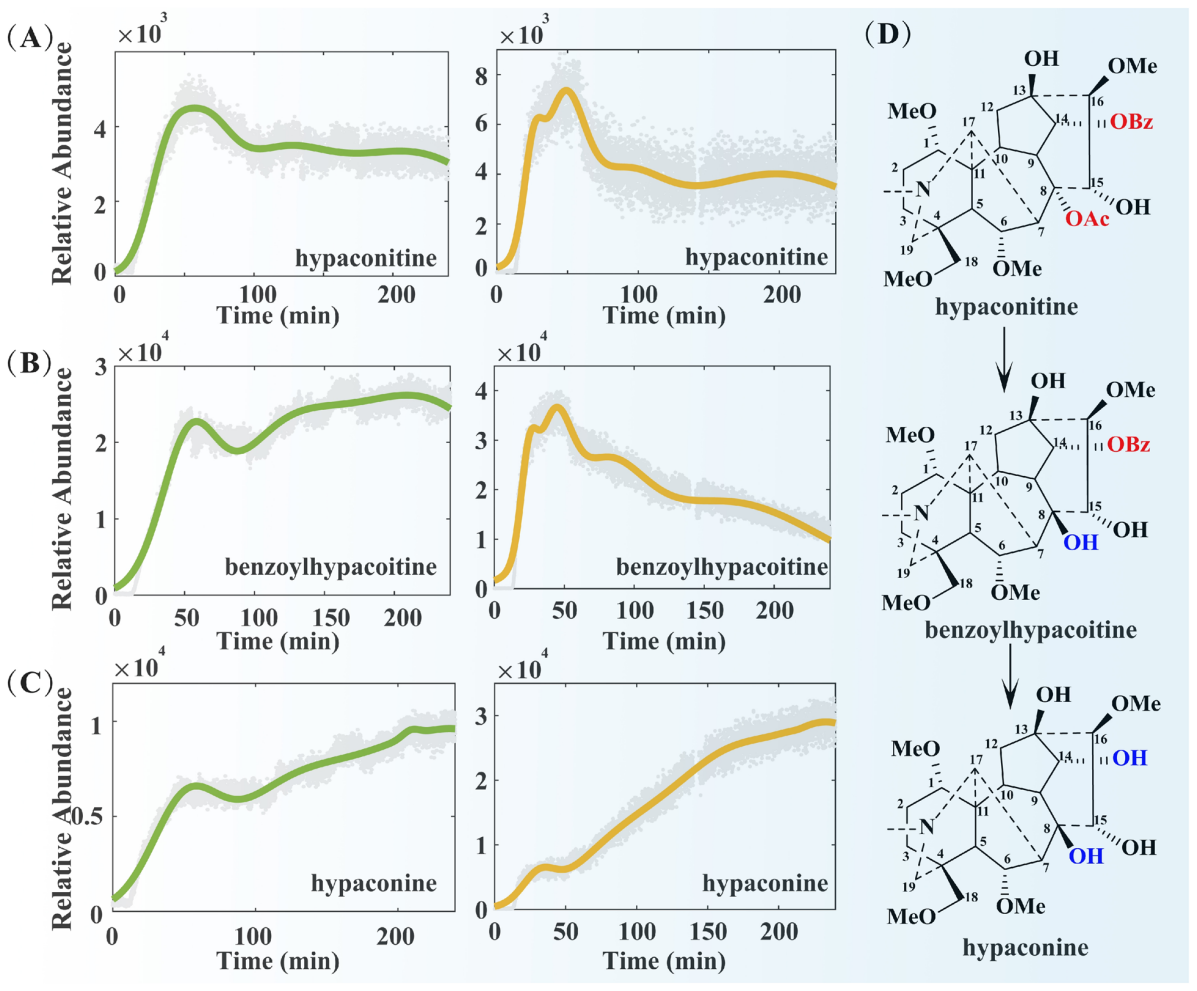
The concentration points and fitting curves of hypaconitine (**A**), benzoylhypacoitine (**B**) and hypaconine (**C**) during decocting

**Fig. 5:**
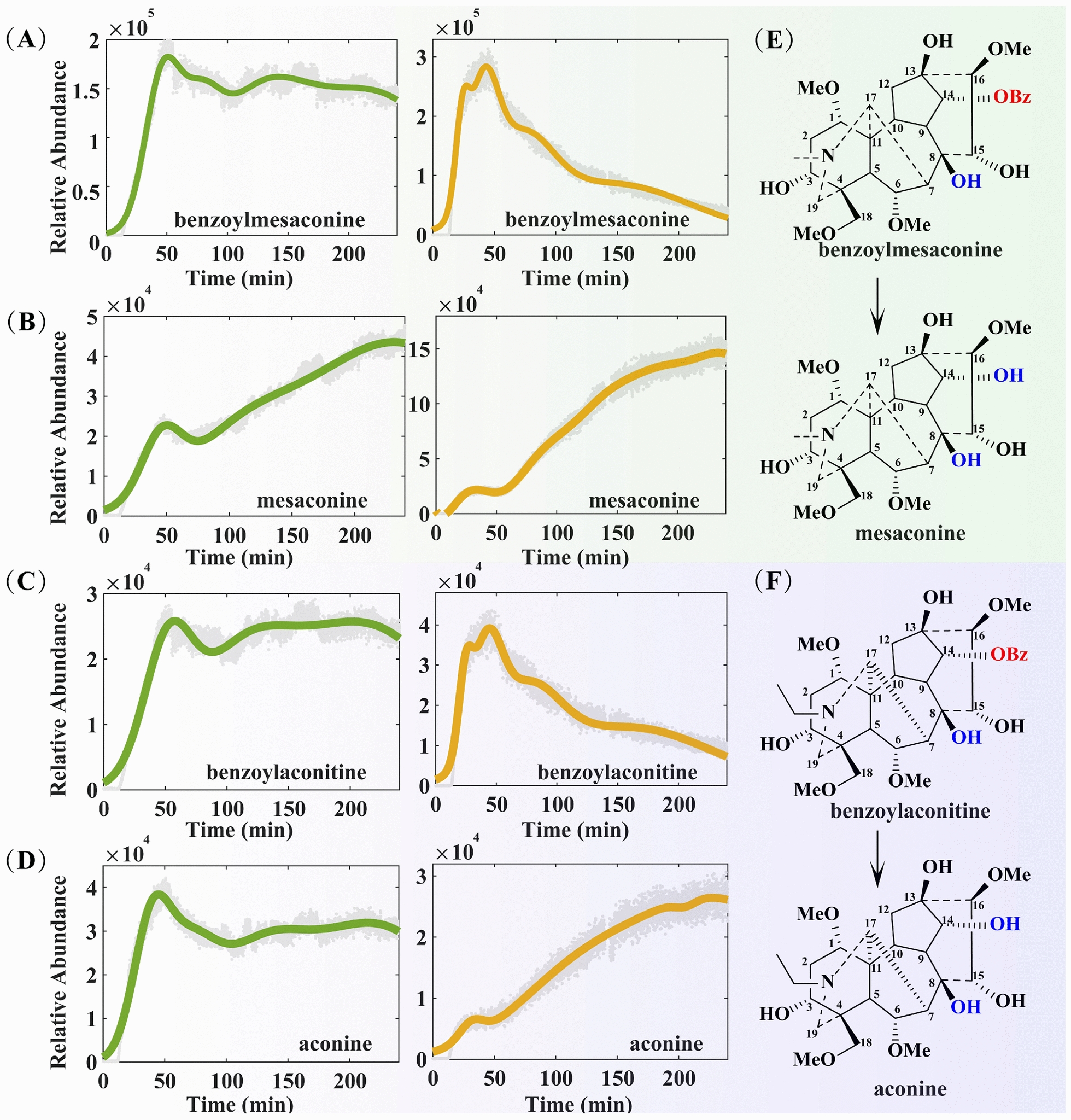
The concentration points and fitting curves of benzoylmesaconine (**A**), mesaconine (**B**), benzoylaconitine (**C**) and aconine (**D**) during decocting

By analyzing the change curves of alkaloid signal intensity in Figs. 4, 5, and S1, we can systematically understand the dynamic evolution patterns of components in the single-decoction system of Fuzi and the co-decoction system of ZWD (where the green curve represents the co-decoction system and the yellow curve represents the single-decoction system). In both the single-decoction and co-decoction systems, various alkaloids (including diester-type, monoester-type, and aminoalcohol-type alkaloids) such as hypaconitine, benzoylhypaconitine, and aconine exhibit highly distinct curve fluctuation characteristics, serving as core markers for component transformation during Fuzi decoction. From the perspective of decoction time, the dissolution and transformation of alkaloids exhibit stage-specific features in both the single-decoction and co-decoction systems. During the soaking and early decoction stages (0–50 min), a large amount of alkaloids dissolved in both types of decoctions, with their concentrations showing a sharp upward trend—indicating that this stage is the main period for alkaloid dissolution. When the decoction time reached 50 min, the changes in alkaloids in the single-decoction system and co-decoction system showed significant differences. In the single-decoction system of Fuzi, the concentration of the diester-type alkaloid hypaconitine (Fig. 4A) decreased rapidly: its signal intensity dropped from a peak of 7.36 × 10^3^ to 3.48 × 10^3^, representing a decrease of 52.7%. In contrast, in the co-decoction system, the dissolution of hypaconitine was affected by other medicinal materials: its maximum signal intensity was 4.49 × 10^3^, which was only 61% of the peak value in the single-decoction system. The significant decrease in the content of such toxic diester-type alkaloids during decoction provides conclusive experimental evidence and solid theoretical support for the scientific connotation of "attenuating" in traditional decoction processes. In the single-decoction system, the concentrations of monoester-type alkaloids benzoylhypaconitine (Fig. 4B), benzoylmesaconine (Fig. 5A), and benzoylaconitine (Fig. 5C) decreased rapidly: benzoylhypaconitine dropped from an initial 3.66 × 10^4^ to 9.93 × 10^3^ (a decrease of 72.9%); benzoylaconitine decreased from 3.92 × 10^4^ to 7.30 × 10^3^ (a decrease of 81.4%); and benzoylmesaconine exhibited the poorest thermal stability and the fastest degradation rate, falling from an initial 2.84 × 10^5^ to 2.86 × 10^4^ (a significant decrease of 89.9%)—which is consistent with the transformation rule of further hydrolysis of monoester-type alkaloids. In the co-decoction system of ZWD, however, the decrease rates of the monoester-type alkaloids were significantly reduced, and some components even showed a concentration rebound: benzoylhypaconitine increased from an initial 2.27 × 10^4^ to 2.45 × 10^4^ (a growth rate of approximately 7.9%, which is presumably related to the inhibitory effect of other medicinal components in the co-decoction system on its hydrolysis or the improved efficiency of its transformation from diester-type alkaloids); benzoylaconitine decreased from an initial 2.58 × 10^4^ to 2.33 × 10^4^ (a degradation rate of only 9.7%); and benzoylmesaconine dropped from an initial 1.83 × 10^5^ to 1.39 × 10^5^ (a decrease of only 24.0%). This further suggests that the compatibility of ZWD can inhibit the hydrolysis of monoester-type alkaloids, promote the retention of some components, and alter the content of cardiotonic active components in Fuzi—verifying the scientific mechanism of "preserving efficacy" in herbal medicine compatibility.

With respect to alcoholamine-type alkaloids, their content changes are directly associated with the hydrolytic transformation of monoester-type alkaloids. In the single-decoction system, hypaconine, mesaconine, and aconine exhibit a sharp upward trend in content due to the targeted hydrolysis of their corresponding monoester-type precursors (benzoylhypacoitine, benzoylmesaconine, and benzoylaconitine). As the most rapidly generated component among alcoholamine-type alkaloids, the signal intensity of mesaconine increased from 2.18 × 10^4^ at 30 min to a peak of 1.46 × 10^5^ at 230 min, representing an increase of up to 570%. Calculations show that this increase is approximately 1.5 times that of hypaconine (345%) and aconine (303%)—a phenomenon consistent with the characteristic of its precursor, benzoylmesaconine, having the fastest degradation rate. In the co-decoction system of ZWD, however, aconine exhibits a unique pattern of content change: within the 50–100 min interval, its content showed a decreasing trend due to the complex effects of other medicinal components in the system (e.g., reversible binding with aconine or the existence of competitive transformation pathways), and tended to stabilize after 100 min. In contrast, the change trends of hypaconine and mesaconine were consistent with those in the single-decoction system. This result indicates that different alcoholamine-type alkaloids are affected by compatibility to significantly varying degrees. In summary, the co-decoction system of ZWD regulates the hydrolysis process of monoester-type alkaloids, reducing the excessive hydrolysis of monoester-type alkaloids into alcoholamine-type alkaloids. This not only reduces the accumulation of toxic substances but also indirectly promotes the reasonable retention of monoester-type alkaloids, thereby achieving the compatibility effect of “toxicity attenuation and efficacy preservation”.

Besides the alkaloids that exhibited significant dynamic changes in the co‑decoction and single‑decoction systems, other alkaloid components in Fig. S1, such as fuzitine and songorine, showed highly similar concentration data points and fitting curves between the two systems. We hypothesize that the fundamental reason for this phenomenon is the relatively high stability of these alkaloids in the decoction system. Under heating conditions, they hardly undergo hydrolysis, intermolecular interactions, or structural transformation. Meanwhile, the chemical constituents from other medicinal herbs in the ZWD co‑decoction system did not exert obvious binding or competitive reactions with these alkaloids, nor did they significantly interfere with their dissolution and existing states. Therefore, the dynamic changes in the content of these alkaloids were only governed by the initial dissolution process, and no significant difference was observed between the co‑decoction and single‑decoction systems. This also reflects the structural diversity of Fuzi alkaloids and their distinct response characteristics to compound compatibility and decoction conditions.

#### Dynamic changes of the main components in Baishao during the decoction process

**Fig. 6:**
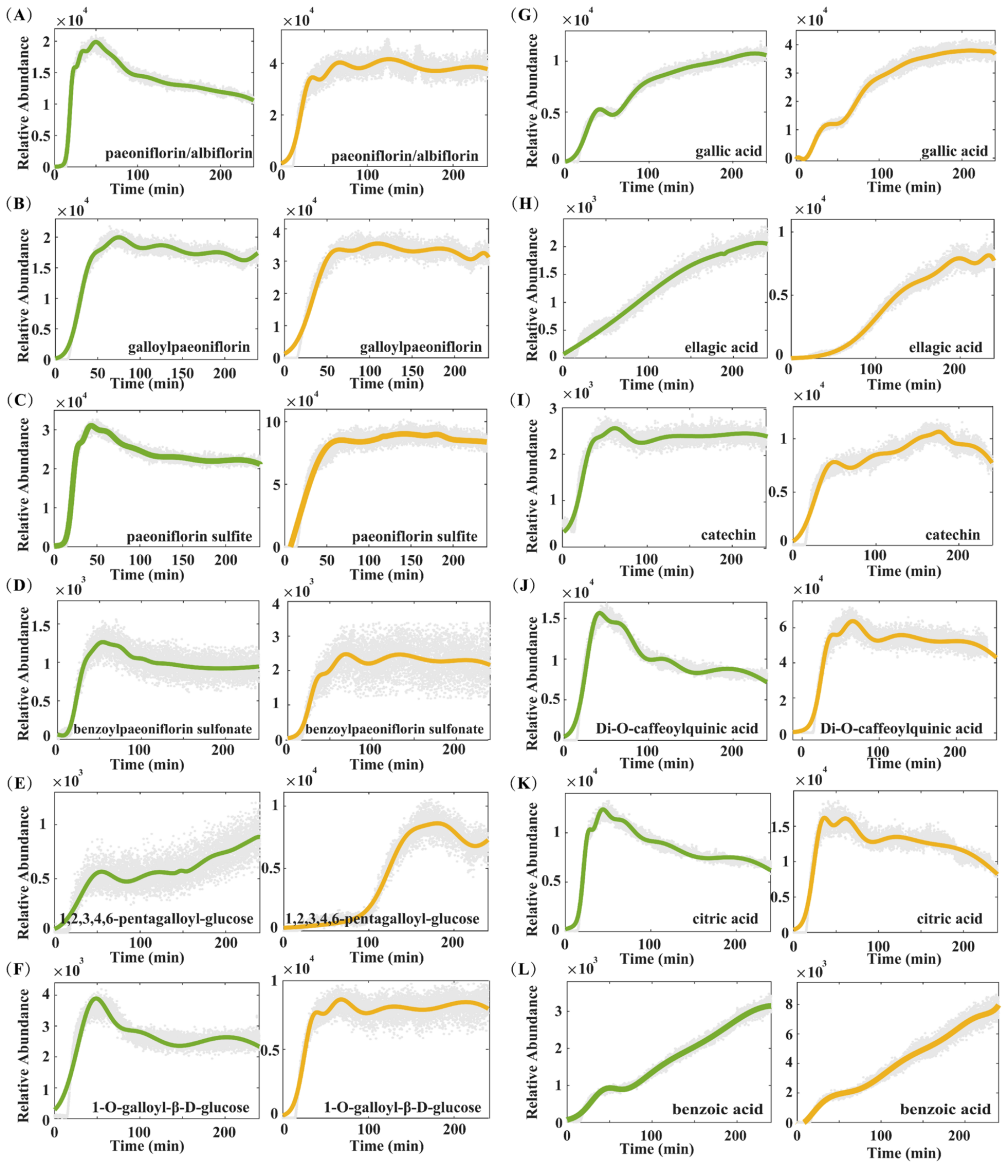
The concentration points and fitting curves of components during the decoction process of Baishao

In the pharmacodynamic system of ZWD, Baishao serves as one of the core components. Its pharmacological effects mainly rely on the synergistic actions of multiple active components, with monoterpenes and their glycosides, as well as polyphenolic compounds, being the primary active constituents. Research has demonstrated that monoterpene components—such as paeoniflorin and albiflorin—exhibit significant anti-inflammatory and analgesic activities: they not only effectively alleviate pain, but also regulate the immune function of body [23, 24]; Polyphenolic components, including gallic acid, 1-O-galloyl-β-D-glucose, and 1,2,3,4,6-penta-galloyl-glucose (among others), exhibit strong antioxidant activity. They can effectively scavenge free radicals and alleviate the damage to cells induced by oxidative stress [25].​ Since the compatibility of herbal medicine affects the existence states of components, the dissolution and transformation behaviors of the aforementioned active components exhibit significant differences between the single-decoction of Baishao and the co-decoction system of ZWD, with the specific details presented below (Figs. 6; S2).

Among monoterpenes and their derivatives, the total signal intensities of paeoniflorin and albiflorin exhibited virtually no significant variation in the single-decoction system of Baishao, but showed a declining trend in the co-decoction system. By 240 min, their total signal intensities had declined by nearly 50% (Fig. 6A). Components such as galloylpaeoniflorin, paeoniflorin sulfonate, and benzoylpaeoniflorin sulfonate (Fig. 6B–D) exhibited a similar trend to paeoniflorin and albiflorin. However, the rate of decline was relatively slower in the co-decoction system. This indicates that different monoterpenes and their glycosides differ in their interactions with other constituents during co-decoction, leading to variations in the rates of their degradation or transformation.

The tannin 1,2,3,4,6-penta-galloyl-glucose is formed by ester-bond linkage of one glucose molecule to five gallic acid molecules, with a relatively complex variation pattern observed during decoction (Fig. 6E). In the single-decoction system of Baishao, the signal intensity of this tannin (1,2,3,4,6-penta-galloyl-glucose) reached 1.92 × 10^3^ at 100 min, with a relatively slow increase rate. This stage is thought to be attributed to the dissolution of herbal tissues and the hydrolysis of other highly substituted galloylglucoses. In contrast, from 100 to 180 min, the increase rate accelerated, and the signal intensity reached 8.62 × 10^3^, this is speculated to result from the intensified hydrolysis of other highly substituted galloylglucoses as decoction progressed, which promoted greater production of this component. Subsequently, a decreasing trend was observed after 180 min, with the signal intensity dropping to 6.80 × 10^3^ at 225 min. This decline is likely because, under sustained heating, the rate at which this component was further hydrolyzed into low-substituted galloylglucoses surpassed the rate at which it was generated via the decomposition of highly substituted galloylglucoses, ultimately leading to a gradual reduction in its content. For another tannin component, 1-O-galloyl-β-D-glucose, its total signal intensity showed almost no significant change in the single-decoction system of Baishao. Yet in the co-decoction system, its signal intensity exhibited a significant decreasing trend, with a reduction of nearly 50% by 240 min (Fig. 6F). This difference may stem from the formation of complexes between this component and other herbal components in the co-decoction system, or its involvement in acyl transfer reactions with aglycone components—both of which lead to a reduction in the content of its free form in the solution.

As hydrolysis products of tannin components, gallic acid and ellagic acid showed a continuous upward trend in both the single-decoction and co-decoction systems. By 240 min, their signal intensities rose to 3.79 × 10^4^ and 8.15 × 10^3^ respectively in the single-decoction system, compared to 1.08 × 10^4^ and 2.09 × 10^3^ respectively in the co-decoction system (Fig. 6G, H). The increase in the early stage of decoction was mainly attributed to the gradual dissolution of herbal tissue, while in the later stage, it may be associated with the hydrolysis of other polysubstituted galloyl compounds such as 1,2,3,4,6-pentagalloylglucose. Catechin remained stable in the co-decoction system, whereas in the single-decoction system, it exhibited a slight increase in the mid-to-late stages followed by a decrease (Fig. 6I). It is hypothesized that specific components in the co-decoction system exert a protective effect on catechin, thereby inhibiting its transformation or degradation. In contrast, the absence of such protective components in the single-decoction system results in an initial increase from the accumulation of dissolved catechin, followed by a content decrease in the later stage as structural transformation or decomposition occurs during prolonged decoction. In the single-decoction system, di-O-caffeoylquinic acid (Fig. 6J) and the carboxylic acid compound citric acid (Fig. 6K) were completely dissolved within 0–50 min and then decreased continuously (with a reduction rate of 26.79% and 48.62% respectively). In contrast, their decrease was more rapid in the co-decoction system, where the reduction rates reached 53.6% and 49.39% respectively. It is inferred that other components in the co-decoction system accelerate their degradation or transformation, or form complexes with them. Benzoic acid exhibited a sharp upward trend in both the co-decoction (238% increase) and single-decoction (309% increase) systems (Fig. 6L). This trend is presumably driven by the hydrolysis of benzoyl-containing substances, which release benzoyl groups that subsequently convert to benzoic acid.

Overall, during the decoction of the compound prescription, on one hand, monoterpene glycosides such as paeoniflorin may react with other herbs to generate new active compounds; on the other hand, the decoction process elevates the content of polyphenolic substances in the system. These two aspects synergistically enhance the therapeutic efficacy, thereby collectively strengthening the antioxidant and immunomodulatory functions of the compound prescription.

#### Dynamic changes of the main components in Shengjiang, Baizhu and Fuling during the decoction process

In studies on the chemical components of ZWD, the three herbs (Shengjiang, Baizhu, and Fuling) have relatively fewer types and lower levels of detectable bioactive components when compared with Fuzi and Baishao. These bioactive components can be roughly divided into two categories: First are the gingerol-type components unique to Shengjiang. As the key material basis for Shengjiang to exert its warming and dredging effects, these components mainly include 6-gingerol, 8-gingerol; Second are the atractylenolide-type components, the signature components of Baizhu, which serve as the main bioactive substances for Baizhu’ s spleen-invigorating and qi-tonifying effects. For Fuling, no characteristic bioactive components were detected, primarily due to the poor water solubility of most of its constituents.

**Fig. 7:**
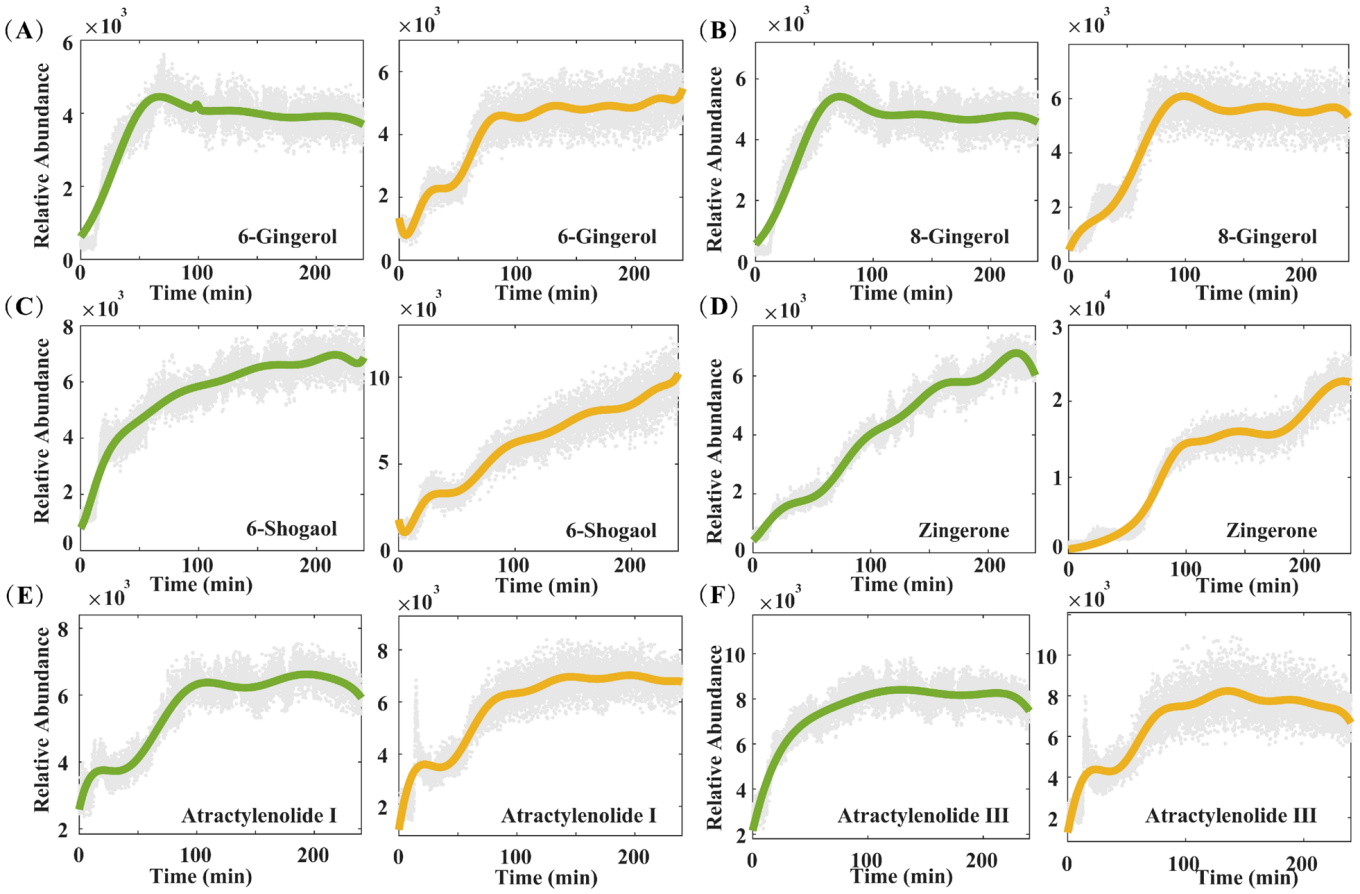
The concentration points and fitting curves of components during the decoction process of Shengjiang (**A**–**D**) and Baizhu (**E**, **F**)

Monitoring of the ginger decoction process showed that the content changes of 6-gingerol and 8-gingerol varied with different systems. Specifically, both components showed a decreasing trend in the co-decoction system, while their contents had no significant change in the single-decoction system. The signal intensities of 6-shogaol and zingerone increased in both systems. For 6-shogaol, its signal intensity in the single-decoction system rose from 3.42 × 10^3^ to 1.03 × 10^4^, with an increase rate of approximately 201.2%, which was about 4 times that of the co-decoction system (51.5%). For zingerone, its signal intensity in the single-decoction system increased from 3.32 × 10^3^ to 2.26 × 10^4^, with a substantial increase rate of 580.7%; in the co-decoction system, it rose from 1.87 × 10^3^ to 6.78 × 10^3^, with an increase rate of approximately 262.6%, and the increase rate in the single-decoction system was about 2.2 times that of the co-decoction system (Fig. 7A–D). Previous studies have confirmed that gingerols can be converted into shogaols under high-temperature conditions [26], which suggests that the co-decoction system may inhibit this conversion process. In addition, monitoring of the atractylenolide components in Baizhu revealed that these components showed a trend of rapid dissolution in the early stage of decoction. After complete dissolution, no significant changes in their contents were observed in either the ZWD co-decoction system or the Baizhu single-decoction system during the subsequent 180-min continuous decoction. This result indicates that atractylenolide components have strong thermal stability, are less likely to interact with components of other herbs in ZWD, and their efficacy mainly depends on sufficient dissolution in the early stage (Fig. 7E, F).

### The proposal of the SBTEF schema for decoction endpoint prediction and the evaluation of ZWD's optimal decoction endpoint

**Fig. 8:**
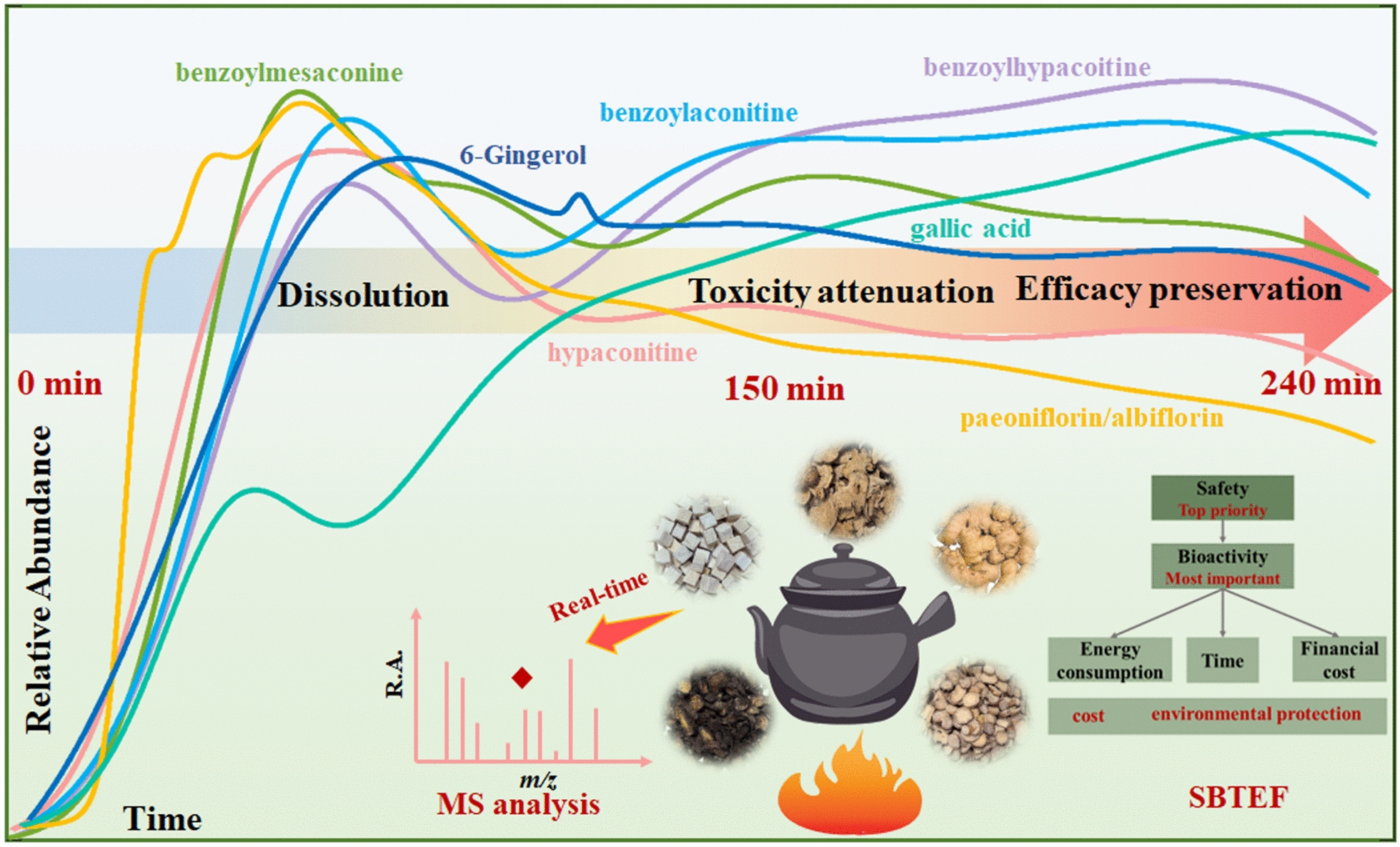
Diagram for predicting the decoction endpoint of ZWD

The complex changes of substances during the decoction of herbal compound prescriptions lead to the difficulty in determining their optimal decoction endpoint. Therefore, in traditional practice, rough determination is mostly based on fixed time or sensory experience. Overall, the ideal goal is to retain active ingredients at a high level to the greatest extent, with the premise that the compound’s safety is guaranteed via the degradation of toxic components. Meanwhile, key factors in practical production and daily life, such as time, cost, and energy consumption, should also be comprehensively considered. Thus, this study first creatively proposes the SBTEF five-dimensional prediction schema for the optimal decoction endpoint of herbal medicines (Fig. 8). Safety is the top priority and fundamental prerequisite, serving as the primary decision‑making criterion for the decoction process, which must ensure that toxic components are degraded to levels below the safety threshold. Bioactivity is critical for guaranteeing therapeutic efficacy. On the premise of meeting safety requirements, efforts should be made to retain key bioactive components (e.g., monoester‑type alkaloids and paeoniflorin) at as high a level as possible to ensure the optimal therapeutic efficacy of the TCM. Considering cost and environmental protection, once safety and bioactivity are satisfied, the optimal decoction endpoint is selected based on the shortest duration, minimal energy consumption, and lowest financial cost. This integrated strategy achieves the dual goals of “reducing toxicity and enhancing efficacy” while optimizing resource utilization.

Following the above logic, ZWD is used as an example. Through a systematic analysis of the dynamic changes in the chemical components of each herb during ZWD’ s decoction, the prediction of its decoction endpoint must be guided by the core principles of “toxicity attenuation and efficacy preservation” as well as the overall synergistic effect of the compound prescription. The transformation rule of Fuzi is the key to determining the decoction time: After approximately 100–150 min of decoction, the diester-type alkaloids (e.g., hypaconitine) are fully hydrolyzed to a low level, and their toxicity is significantly reduced. In contrast, the core efficacy-related monoester-type alkaloids (e.g., benzoylhypacoitine) benefit from the hydrolysis-inhibiting effect of the compatibility environment in the co-decoction system, maintaining a relatively stable plateau in concentration between 100 and 150 min. Therefore, the decoction time for Fuzi should be controlled within 100–150 min. The active components of Baishao support a slightly longer decoction time: Its main glycoside components (e.g., paeoniflorin) undergo slow transformation, while the hydrolysis of polyphenolic components (e.g., 1,2,3,4,6-penta-galloyl-glucose) and the production of its active metabolites (gallic acid, ellagic acid) are more active in the middle and late stages of decoction (100–240 min). A longer decoction time is required to achieve the balance of dissolution and transformation, thereby enhancing antioxidant activity. During decoction, the content of shogaols in Shengjiang shows a continuous upward trend, while the atractylenolides in Baizhu dissolve out in the early stage and then remain stable. Both support medium-to-long-term decoction. In conclusion, under the present experimental conditions and dosage employed in this study, to balance the needs of “toxicity attenuation and efficacy preservation” for Fuzi, as well as the requirements for component dissolution and transformation of Baishao and Shengjiang, it is most appropriate to set the decoction endpoint of ZWD at approximately 150 min. This time point not only ensures the effective removal of toxic components and the optimal retention of efficacy components in Fuzi but also promotes the sufficient hydrolysis of polyphenolic components in Baishao and the production of active metabolites, thereby maximizing the overall efficacy of the compound prescription (Fig. 8). This endpoint prediction provides a quantitative scientific basis for the traditional decoction process of ZWD and achieves the unification of toxicity attenuation and efficacy preservation.

## Conclusion and future directions

Based on the self-established ORT-MS technical platform, this study develops a universal real-time analysis strategy for the decoction process of herbal medicine, and proposes a five-dimensional collaborative prediction schema for the endpoint of decoction that integrates safety, bioactivity, time, energy consumption, and financial cost. Taking ZWD as a research paradigm, this strategy successfully captures the dynamic evolution characteristics of 51 key components (including alkaloids, monoterpene glycosides, etc.) in different decoction stages in real time, clarifies the compatibility mechanism of “toxicity attenuation and efficacy preservation”, and determines 150 min as the optimal decoction endpoint combined with the five-dimensional schema SBTEF. This ensures the maximization of the overall efficacy of the compound prescription and provides an important tool for the transformation of herbal medicine decoction from experience-driven to scientific standardization.

However, this study still has certain limitations: some low-abundance and poorly water-soluble components were not detected, which may affect the comprehensiveness of dynamic analysis of components in the decoction system; isotopic and isomeric interferences exist, leading to a relatively high risk of component interference; the optimal endpoint determined based on in vitro simulated decoction has not been verified by animal or clinical studies regarding its correlation with the in vivo efficacy, toxicity, and metabolic characteristics of ZWD. Thus, its clinical translational value remains to be explored. To address the above shortcomings, future research directions are proposed: relying on the strong universality of the ORT-MS, coupling it with high-resolution mass spectrometry (HRMS) and ion mobility spectrometry (IMS) to reduce isotopic and isomeric interference and further improve detection accuracy; conducting in vivo animal and clinical efficacy studies to verify the effectiveness and safety of the 150 min decoction endpoint of ZWD; and establishing a comprehensive evaluation system for decoction endpoints integrating in vitro component analysis and in vivo pharmacological and toxicological evaluation.

## Supplementary Information

Supplementary material 1.

## Data Availability

No datasets were generated or analysed during the current study.

## Abbreviations

B: Bioactivity
E: Energy consumption
F: Financial cost
ORT-MS: Online Real-Time Mass Spectrometry
S: Safety
T: Time
ZWD: Zhenwu decoction
