# Supplementary material for: Online real-time mass spectrometry-driven dynamic monitoring and optimal endpoint prediction schema development for herbal medicine decoction process
Source: Chin Med. 2026 Mar 16;21:84. doi: 10.1186/s13020-026-01377-1 (PMC12990406; doi:10.1186/s13020-026-01377-1)
Supplement: Supplementary file 1 — Supplementary material 1. [file 13020_2026_1377_MOESM1_ESM.docx]

**Table S1** Components identified from ZWD by ORT-MS.

| **No.** | **m/z** | **Fragments** | **Adduct ions** | **Formula** | **Identity** | **Ref.** |
| --- | --- | --- | --- | --- | --- | --- |
| 1 | 342 | 297, 279, 265 | [M+H]^+^ | C_20_H_23_NO_4_ | fuzitine | [1] |
| 2 | 358 | 340, 322 | [M+H]^+^ | C_22_H_31_NO_3_ | songorine | [2] |
| 3 | 360 | 342, 324 | [M+H]^+^ | C_22_H_33_NO_3_ | napelline | [2] |
| 4 | 378 | 360, 342, 332, 328 | [M+H]^+^ | C_22_H_35_NO_4_ | karakoline | [2] |
| 5 | 394 | 376, 358, 340, 328 | [M+H]^+^ | C_22_H_35_NO_5_ | chuanfumine | [1] |
| 6 | 408 | 390, 358 | [M+H]^+^ | C_23_H_37_NO_5_ | Cammaconine | [2] |
| 7 | 422 | 404, 390, 372,358 | [M+H]^+^ | C_24_H_39_NO_5_ | talatizamine | [1] |
| 8 | 438 | 420, 388, 356 | [M+H]^+^ | C_24_H_39_NO_6_ | neoline | [1] |
| 9 | 454 | 436, 404, 386, 372 | [M+H]^+^ | C_24_H_39_NO_7_ | fuziline | [1] |
| 10 | 470 | 438, 406, 356 | [M+H]^+^ | C_24_H_39_NO_8_ | hypaconine | [2] |
| 11 | 468 | 450, 436, 418 404, 386, 372, 354 | [M+H]^+^ | C_25_H_41_NO_7_ | delsoline | [2] |
| 12 | 486 | 468, 454, 436, 422, 404, 390 | [M+H]^+^ | C_24_H_39_NO_9_ | mesaconine | [2] |
| 13 | 498 | 466, 448 | [M+H]^+^ | C_29_H_39_NO_6_ | delavaconitine | [2] |
| 14 | 558 | 540,508 | [M+H]^+^ |  | 14-benzoyl-deoxyhypaconine | [2] |
| 15 | 500 | 482, 468, 450, 436, 418, 404, 392 | [M+H]^+^ | C_25_H_41_NO_9_ | aconine | [2] |
| 16 | 574 | 542, 510, 492, 478 | [M+H]^+^ | C_31_H_43_NO_9_ | benzoylhypacoitine | [2] |
| 17 | 588 | 524, 556 | [M+H]^+^ | C_32_H_45_NO_9_ | benzoyldeoxyaconine | [2] |
| 18 | 590 | 572, 558, 558, 540, 526, 508, 494, 476 | [M+H]^+^ | C_31_H_43_NO_10_ | benzoylmesaconine | [2] |
| 19 | 604 | 586, 572, 554, 540, 522, 496 | [M+H]^+^ | C_32_H_45_NO_10_ | benzoylaconitine | [2] |
| 20 | 606 | 588, 574, 556, 542, 524 | [M+H]^+^ | C_31_H_43_NO_11_ | 14-benzoyl-10-OH-mesaconine | [2] |
| 21 | 616 | 584, 556 | [M+H]^+^ | C_33_H_45_NO_10_ | hypaconitine | [2] |
| 22 | 616 | 598, 584, 566, 552, 534 | [M+H]^+^ | C_33_H_45_NO_10_ | pyrojesaconitine | [2] |
| 23 | 700 | 640, 578, 500, 414 | [M+H]^+^ | C_39_H_41_NO_11_ | trifoliolasine E | [2] |
| 24 | 169 | 125 | [M-H]^-^ | C_7_H_6_O_5_ | gallic acid | [4] |
| 25 | 191 | 173, 111 | [M-H]^-^ | C_6_H_8_O_7_ | citric acid | [5] |
| 26 | 289 | 245, 179, 151, 137, 109 | [M-H]^-^ | C_15_H_14_O_6_ | catechin | [5] |
| 27 | 301 | 284, 229, 185 | [M-H]^-^ | C_14_H_6_O_8_ | ellagic acid | [6] |
| 28 | 331 | 211, 169, 151, 125 | [M-H]^-^ | C_13_H_16_O_10_ | 1-O-galloyl-β-D-glucose | [5] |
| 29 | 341 | 179, 161 | [M-H]^-^ | C_12_H_22_O_11_ | sucrose | [5] |
| 30 | 421 | 375, 345, 165 | [M+HCOO]^-^ | C_16_H_24_O_10_ | debenzoylpaeoniflorin | [7] |
| 31 | 493 | 331, 313, 169 | [M-H]^-^ | C_19_H_26_O_15_ | 6-O-galloylsucrose | [4] |
| 32 | 515 | 353 | [M-H]^-^ | C_25_H_24_O_12_ | di-O-caffeoylquinic acid | [8] |
| 33 | 525 | 449, 327 | [M+HCOO]^‒^ | C_23_H_28_O_11_ | paeoniflorin/albiflorin | [4] |
| 34 | 543 | 497, 421, 259, 213 | [M-H]^-^ | C_23_H_28_O_13_S | paeoniflorin sulfite | [7] |
| 35 | 631 | 613, 313 | [M-H]^-^ | C_30_H_32_O_15_ | galloylpaeoniflorin | [4] |
| 36 | 647 | 525, 259, 479 | [M-H]^-^ | C_30_H_32_O_14_S | benzoylpaeoniflorin sulfonate | [7] |
| 37 | 787 | 635, 617, 465, 295 | [M-H]^-^ | C_34_H_28_O_22_ | tetra-O-galloylglucose | [4] |
| 38 | 939 | 769, 617, 447 | [M-H]^-^ | C_41_H_32_O_26_ | 1,2,3,4,6-penta-galloyl-glucose | [4] |
| 39 | 121 | / | [M-H]^-^ | C_7_H_6_O_2_ | benzoic acid | / |
| 40 | 559 | 541, 515, 471 | [M-H]^-^ | C_33_H_52_O_7_ | 25-Methoxy-29-hydroxyporicoic acid HM | [9] |
| 41 | 515 | 497, 441 | [M-H]^-^ | C_31_H_47_O_6_ | 16alpha,29-dihydroxy-3,4-seco-lanosta-4(28),8,24(31)-trien-3,21-dioic acid | [10] |
| 42 | 497 | 479, 435, 419, 309 | [M-H]^-^ | C_31_H_46_O_5_ | 16α,25‐dihydroxydehydroeburiconic acid | [9] |
| 43 | 453 | 391 | [M-H]^-^ | C_30_H_46_O_3_ | dehydrotrametenolic acid | [9] |
| 44 | 175 | 130, 116 | [M+H]^+^ | C_6_H_14_N_4_O_2_ | L (+)-arginine | [11] |
| 45 | 231 | 203, 185, 163, 105 | [M+H]^+^ | C_15_H_18_O_2_ | atractylenolide Ⅰ | [1] |
| 46 | 233 | 215, 187, 131, 105 | [M+H]^+^ | C1_5_H_20_O_2_ | atractylenolide Ⅱ | [1] |
| 47 | 249 | 231, 213, 189 | [M+H]^+^ | C_15_H_20_O_3_ | atractylenolide Ⅲ | [1] |
| 48 | 195 | 177, 145, 137, 117 | [M+H]^+^ | C_11_H_14_O_3_ | zingerone | [1] |
| 49 | 295 | 277, 137 | [M+H]^+^ | C_17_H_26_O_4_ | 6-gingerol | [1] |
| 50 | 323 | 305, 137 | [M+H]^+^ | C_19_H_30_O_4_ | 8-gingerol | [1] |
| 51 | 277 | 137 | [M+H]^+^ | C_17_H_24_O_3_ | 6-shogaol | [1] |


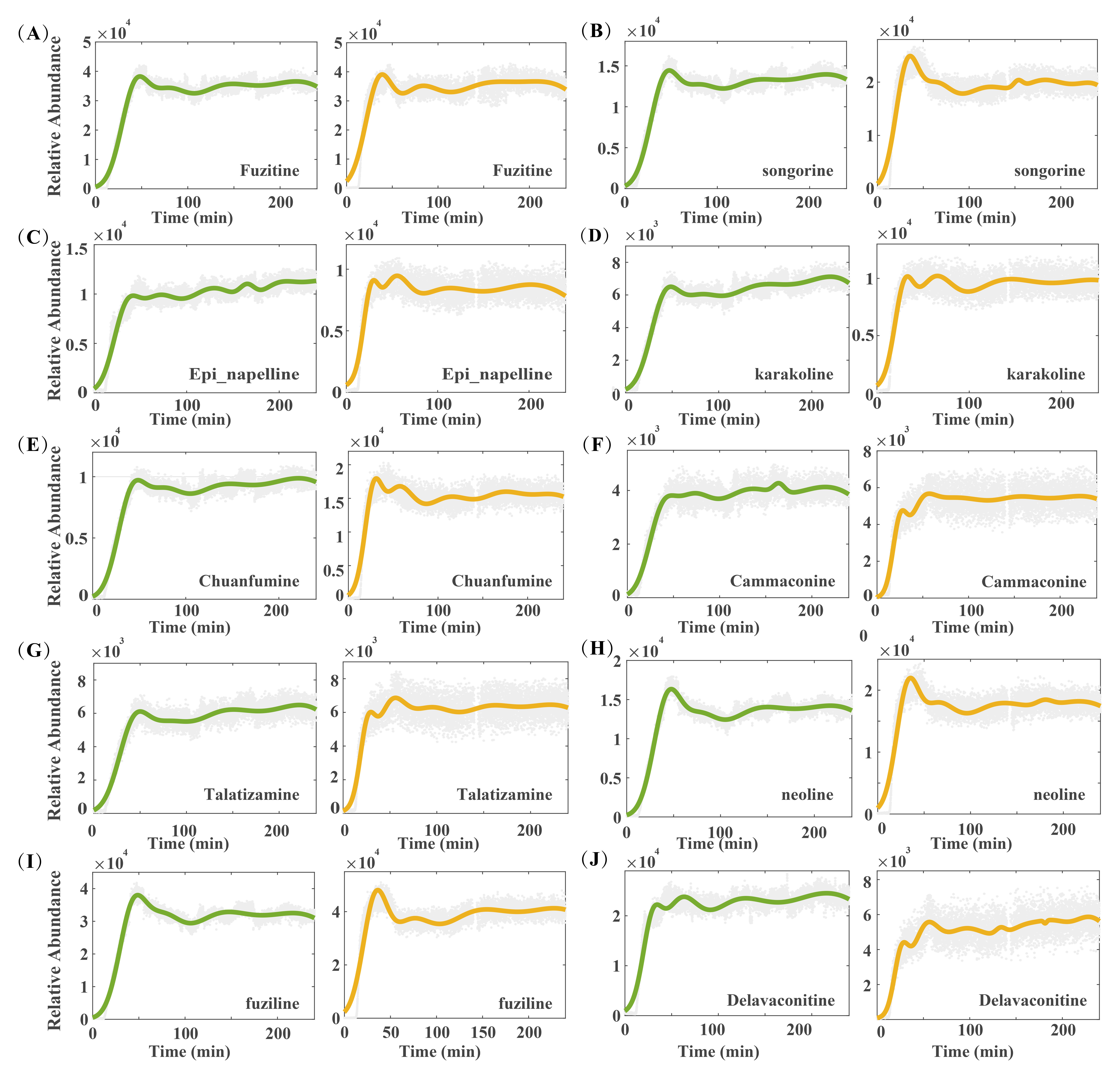


**Fig. S1.** The concentration points and fitting curves for other components during the decoction process of Fuzi.


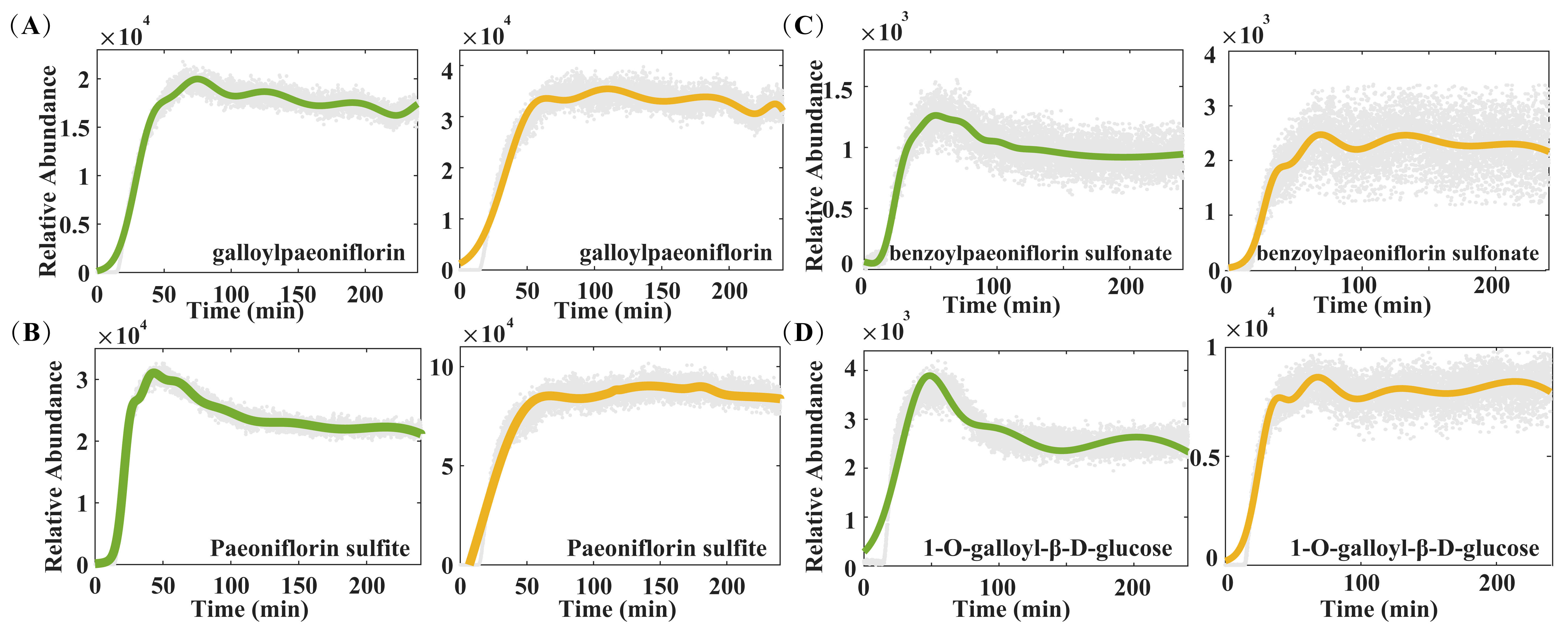


**Fig. S2.** The concentration points and fitting curves for other components during the decoction process of Baishao.

**References**

[1] Su YX, Shen LY, Zhu PX, Liang XR. UPLC-ESI-Q-TOF-MS/MS based metabolomics investigation on chemical constituent consistency of Zhenwu Decoction before and after compatibility. J. Pharmaceut. Biomed. 2024; 246: 116222.

[2] Xu F L, Zhang QY, Peng G., Yao R, Li BT, Xu GL. Chemical composition analysis of Radi*x Aconiti Lateralis Preparata* by UHPLC-Q-TOF/MS. Jiangxi J. Tradit. Chin. Med. 2015; 46(2): 48-52.

[3] Qiu ZD, Wei XY, Sun RQ, Chen JL, Tan T, Xu JQ, et al. Limitation standard of toxic aconitines in Aconitum proprietary Chinese medicines using on-line extraction electrospray ionization mass spectrometry. Acta Pharm. Sin. B. 2020;10: 1511-1520.

[4] Xu C, Wang X, Han J, Gu Z, Guo Q. LMD and LC-MS-based chemical constituents and pharmacological effects assessment for two different processing methods of the root of *Paeonia lactiflora* Pall. J. Pharm. Biomed. Anal. 2024; 245:116184.

[5] Xu Y, Cai H, Cao G, Duan Y, Pei K, Tu S, et al. Profiling and analysis of multiple constituents in Baizhu Shaoyao San before and after processing by stir-frying using UHPLC/Q-TOF-MS/MS coupled with multivariate statistical analysis. J. Chromatogr. B Analyt. Technol. Biomed. Life Sci. 2018; 1083: 110-123.

[6] Kumar S, Singh A, Kumar B. Identification and characterization of phenolics and terpenoids from ethanolic extracts of Phyllanthus species by HPLC-ESI-QTOF-MS/MS. J Pharm. Anal. 2017; 7(4): 214-222.

[7] Liu J, Chen L, Fan CR, Li H, Huang MQ, Xiang Q, et al. 2015. Qualitative and quantitative study on the main components of white peony root and red peony root based on HPLC-DAD-Q-TOF-MS/MS. China J Chin. Mater. Med. 40; (9): 1762-1770.

[8] Ferracane R, Pellegrini N, Visconti A, Graziani G, Chiavaro E, Miglio C, et al. Effects of different cooking methods on antioxidant profile, antioxidant capacity, and physical characteristics of artichoke. J. Agric. Food Chem. 2008; 56(18): 8601-8608.

[9] Zou YT, Long F, Wu CY, Zhou J, Zhang W, Xu JD, et al. A dereplication strategy for identifying triterpene acid analogues in *Poria cocos* by comparing predicted and acquired UPLC-ESI-QTOF-MS/MS data. Phytochem. Anal. 2019; 30(3): 292-310.

[10] Wang W, Dong H, Yan R, Li H, Li P, Chen P, et al. Comparative study of lanostane-type triterpene acids in different parts of *Poria cocos* (Schw.) Wolf by UHPLC-Fourier transform MS and UHPLC-triple quadruple MS. J. Pharmaceut. Biomed. 2015; 102: 203–214.

[11] Benito S, Sánchez A, Unceta N, Andrade F, Aldámiz-Echevarria L, Goicolea MA, et al. LC-QTOF-MS-based targeted metabolomics of arginine-creatine metabolic pathway-related compounds in plasma: application to identify potential biomarkers in pediatric chronic kidney disease. Anal. Bioanal. Chem. 2016; 408(3): 747-760.
